# Lipid abnormalities in streptozotocin-diabetes: Amelioration by *Morus indica* L. cv Suguna leaves

**DOI:** 10.4103/0973-3930.54289

**Published:** 2009

**Authors:** B. Andallu, A. V. Vinay Kumar, N. Ch. Varadacharyulu

**Affiliations:** Sri Sathya Sai University, Anantapur - 515 001, Andhra Pradesh, India; 1 The Institute of Environmental and Human Health, Texas Tech University, USA; 2 Department of Biochemistry, Sri Krishnadevaraya University, Anantapur, India

**Keywords:** Atherogenic index, bile acids, cholesterol, hyperlipidemia, mulberry leaves, STZ- diabetic rats, triglycerides

## Abstract

**AIM:**

To observe the influence of mulberry (*Morus indica* L. cv Suguna) leaves on lipid abnormalities in STZ-diabetic rats.

**MATERIALS AND METHODS:**

Treatment with dried mulberry leaf powder for a period of 8 weeks in hyperglycemic and hyperlipidemic STZ-diabetic rats.

**RESULTS:**

Mulberry leaves regulated fasting blood glucose, ameliorated the abnormalities in lipid profile as indicated by significant (*P*<0.01) decrease in serum triglycerides, phospholipids, cholesterol and plasma free fatty acids by 50, 6, 31 and 22% respectively in STZ- diabetic rats compared to diabetic control rats which had significantly (*P*<0.01) raised levels of triglycerides, phospholipids, cholesterol and free fatty acids than the normal control rats. A marked increase in fecal bile acids (154%) was observed in mulberry treated diabetic rats compared to the diabetic control group indicating conversion of cholesterol to bile acids. In addition, mulberry supplementation significantly lowered LDL-C (67%) and VLDL-C (44%) levels and increased HDL-C (53%) and also decreased atherogenic index (58%) significantly when compared to the diabetic control group.

**CONCLUSION:**

Besides the diabetic rats, mulberry leaves affected lipid profile in normal rats also indicating hypolipidemic effect as a result of the synergistic action of bioactive compounds.

## Introduction

Diabetes mellitus is associated with a large number of lipid abnormalities. Emerging evidence confirms the pivotal role of hyperlipemia, mainly elevated blood cholesterol, particularly LDL cholesterol and VLDL cholesterol in the development of atherosclerosis–related disease.[1] Significant abnormalities in lipid metabolism and lipoproteins in diabetes are evident which in turn depend on the extent of insulin deficiency, insulin resistance, obesity, diet and the presence of concomitant primary and other secondary causes of hyperlipemia. In diabetic hyperlipemia, a series of bizarre lipoproteins and other lipids appear and interaction of this with oxidative stress and free radicals leads to enhanced lipid peroxidation in plasma, tissues and membranes, causing extensive tissue damage. It is well known that lipid peroxidation provides a continuous supply of free radicals that play an important role in etiopathogenesis of diabetes and its complications.[2] Various therapeutic methods used in diabetes treatment available today achieve transiently regulated euglycemia but fail to prevent lipid and lipoprotein alterations, ultimately, exposing the diabetic humans and animals to cardiovascular complications.[3] Moreover, many of these drugs exert various side/toxic[4] effects such as hepatotoxicity (troglitazone) or cardiac failure [rosiglitazone).[5] Concurrently, phytochemicals identified from traditional medicinal plants are presenting an exciting opportunity for the development of new types of therapeutics. This has accelerated the global effort to harness and harvest those medicinal plants that bear substantial amount of potential phytochemicals showing multiple beneficial effects in combating diabetes and the related complications without causing side effects.[6] In the recent years, search for natural dietary therapeutic methods for controlling diabetes are much active as diet plays a key role in the treatment of diabetes.[7]

Mulberry has been explored as a medicinal plant and its medicinal properties are testified in various scriptures. It occupies an important position in the holistic system of Indian medicine ‘Ayurveda’ which has its roots in antiquity and has been practiced for centuries. The leaves of mulberry are nutritious, palatable, nontoxic and also enriched with different active principles.[8] Mulberry leaves (*Morus alba* L.) have been used to cure “Xiao-ke” (diabetes) in traditional Chinese medicine.[9] Mulberry leaves are used as traditional medicine with anti-inflammatory and antihyperglycemic actions.[10,11] The antihyperglycemic effects of six N-containing sugars present in mulberry leaves were investigated in STZ-induced diabetic mice.[12] The root bark of *Morus alba* L. has been used as a blood pressure depressant in China and Japan from old times[13] but no reports are available on the hypolipidemic effect. Hence, the present research on mulberry opens up a new avenue to develop a novel therapy to combat diabetes and concomitant hyperlipidemia.

## Materials and Methods

Male albino rats (24) of Wistar strain with body weights ranging from 150-200g, procured from National Centre for Laboratory Animal Sciences, National Institute of Nutrition, Hyderabad were housed in individual cages in an air conditioned room (25° ± 1°C) with light from 7 a.m to 7 p.m. The rats were allowed to acclimatize to the laboratory environment for 7 days, distributed into 4 groups according to the similar weights with six animals in each group as given below:

Group I – Normal control

Group II – Normal treated with mulberry leaf powder

Group III – Diabetic control

Group IV – Diabetic treated with mulberry leaf powder

The mulberry leaves (*Morus indica* L.) were procured from the Regional Sericultural Research Station, Raptadu, Anantapur District, washed thoroughly, shade dried, powdered in an electric mixer and used in the experiment.

Animals of group III and IV were rendered diabetic by a single intra-peritoneal injection of streptozotocin (55 mg/kg).[14] Control rats (Group I and II) were injected with citrate buffer alone. After 72 hrs of injection, blood was drawn from retro-orbital plexus of conscious rats using heparinized capillaries and glucose was estimated by glucose oxidase method[15] using Span Diagnostic kit.

Group II and IV received experimental feed containing powdered mulberry leaves at 25% level (as per dose response) mixed with the standard feed (obtained from NCLAS, NIN, Hyderabad, A.P.). Group I and III received standard feed in powder form mixed with cellulose and protein to make it isocaloric to the experimental feed. All the groups of rats were maintained under standard housing conditions for a period of 8 weeks with free access to food and water. After 8 weeks of period, over-night fasted rats were sacrificed by exsanguination and blood was collected by cardiac puncture in vials for analytical procedures. Glucose,[15] cholesterol,[16] triglycerides,[17] phospholipids[18] and HDL cholesterol[19] in serum and free fatty acids[20] in plasma were estimated. LDL and VLDL cholesterol[21] and atherogenic index[22] were calculated. Fecal bile acid content was estimated by the method of Scott.[23] Mean and standard error were calculated.[24] The data were statistically analysed by applying Duncan's Multiple Range Test[25] to assess the significant differences among the groups and values of *P*<0.05 were regarded as significant. These experiments were conducted as per CPCSEA guidelines and were approved by Institutional Ethical Committee.

## Results

Mulberry supplementation significantly decreased fasting glucose (58%), lowered serum triglycerides, phospholipids, cholesterol and plasma free fatty acids by 50, 6, 31 and 22% respectively in diabetic rats compared to control rats which were characterized by significantly raised levels of fasting glucose (274%), triglycerides (144%), phospholipids (16%), cholesterol (42%) and free fatty acids (46%) compared to the normal control rats. A marked increase in fecal bile acids (154%) was recorded in mulberry fed diabetic rats compared to the diabetic control group which showed significant fall in fecal bile acids (22%) compared to the normal control rats [Table 1].

**Table 1:** Serum lipid profile and fecal bile acids in different groups of rats

Parameter	Normal control	Normal mulberry	Diabetic control	Diabetic mulberry
Fasting blood glucose (mg/dl)	98.6±5.2	92.3±6.7 (6)	369.2±4.8**(274)	153.6±2.7**(58)
Triglycerides (mg/dl)	80.9±1.9	73.1±1.7* (9)	197.5±4.3**(144)	98.91±2.4**(50)
Phospholipids (mg/dl)	112.5±0.5	96.5±0.4** (14)	130.2±0.6**(16)	122.3±0.6**(6)
Free fatty acids (mg/dl)	8.9±0.3	4.0±0.2* (55)	13.0±0.3** (46)	10.1±0.3**(22)
Cholesterol (mg/dl)	74.8±0.7	64.5±0.9** (14)	106.4±1.9** (42)	72.9±0.6**(31)
Fecal bile acids (mg/rat/day)	19.5±0.6	34.7±1.3* (78)	15.2±0.7**(22)	38.6±0.5**(154)

Values are mean + SEM of 6 rats in each group, Comparison between groups 1 and 2, 1 and 3 and 3 and 4, Figures in parentheses show percent increase or decrease over respective control

* *P*<0.05;

** *P*<0.01

**Figure 1:**
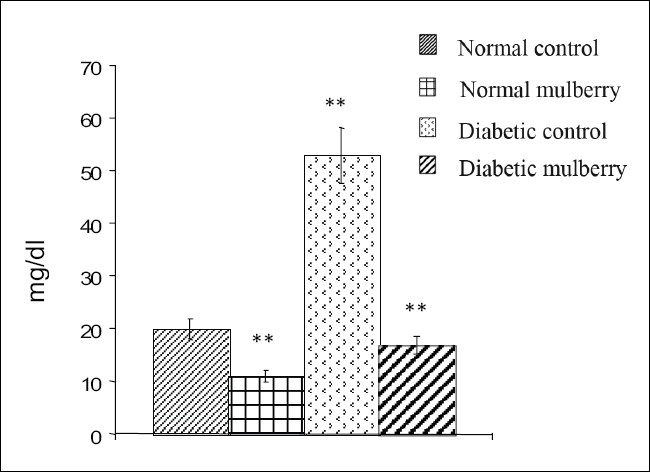
LDL- Cholesterol levels in different groups of rats

**Figure 2:**
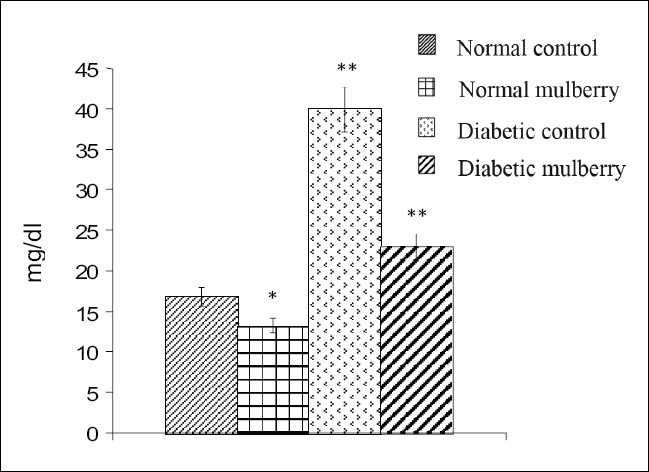
VLDL- Cholesterol levels in different groups of rats

**Figure 3:**
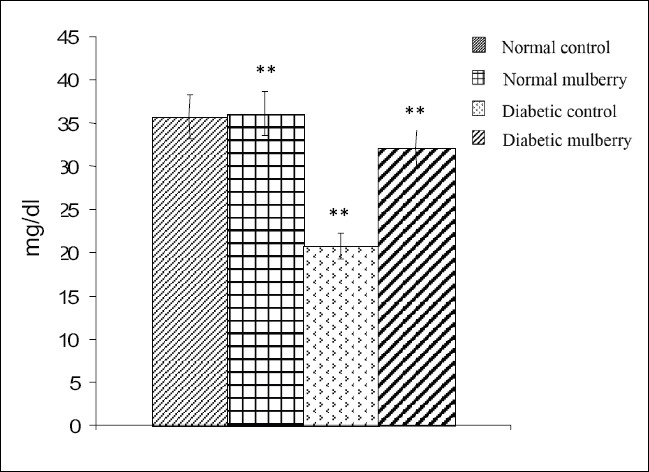
HDL- Cholesterol levels in different groups of rats

**Figure 4:**
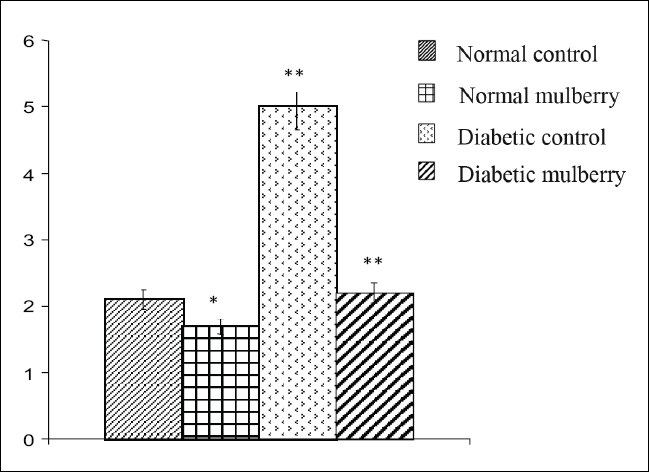
Atherogenic index in different groups of rats

The values of different fractions of cholesterol viz., LDL, VLDL, HDL, and atherogenic index from different groups of rats are furnished in Figures 1–4. Mulberry supplementation significantly lowered LDL-C (68%) and VLDL-C (43%) levels and increased HDL-C (54%) and also decreased atherogenic index (56%) significantly when compared to the diabetic control group which exhibited significant hike in the levels of LDL -C and VLDL-C and atherogenic index by 165, 138 and 138% respectively and decrease in the level of HDL-C by 42% compared to the control group as shown in the Figures 1,2,3 and 4 respectively. Besides diabetic rats, mulberry supplementation influenced almost all the aforesaid parameters in normal rats.

## Discussion

Hyperlipidemia is a known complication of diabetes mellitus[26] and coexists with hyperglycemia and is characterized by increased levels of cholesterol, triglycerides and phospholipids, and also changes in lipoproteins.[27] Interest in the study of plasma lipids in diabetes arises from the widely acknowledged higher incidence of atherosclerotic disease which is a major cause of premature death in diabetic patients.[28]

In the present study, serum triglycerides, phospholipids, cholesterol, LDL cholesterol, VLDL cholesterol and atherogenic index were significantly elevated while HDL cholesterol and fecal bile acids were significantly decreased in diabetic rats. Interestingly, the results further indicated that all these lipid and lipoprotein abnormalities were countered by mulberry leaves in diabetic rats. Glycemic control appears to be the major determinant of total and VLDL triglyceride concentrations.[29]

Control of hyperlipidemia is a prerequisite for the prevention of diabetic microangiopathy (retinopathy, nephropathy and neuropathy) and macroangiopathy (ischemic heart disease), cerebral vascular disease (CVD) and arteriosclerosis obliterans in diabetes.[30] However, in certain cases of diabetes, treatment with insulin with normalization of plasma glucose levels did not restore the HDL-C concentrations to normal implying that, factors in addition to hyperglycemia are causing the lower HDL-C.[31]

Synthesis of VLDL is promoted by an increase in the flux of free fatty acids in liver and ultimately the particles are converted to LDL. Studies revealed increased levels of VLDL as a consequence of decreased clearance and also over-production in type 1 DM subjects. The increased circulatory VLDL-C and the associated triglycerides due to defective clearance[32] of these particles from circulation is in agreement with earlier studies of Babu and Srinivasan[33] and others and these changes were attributed to the altered activity of lipoprotein lipase.

It seems that the changes in adipose tissue lipolysis or intrahepatic mechanisms involving other changes in fractional esterification of fatty acids are in the assembly or secretion of VLDL are responsible for the increase in triacylglycerol secretion rate. *In vitro* studies have shown a decrease in fractional catabolic rate for LDL from type 2 DM subjects and also evidence suggests that *in vivo* nonenzymatic glycosylation of LDL may result in decreased LDL clearance.[34] LDL cholesterol concentrations are strongly and positively related to atherosclerotic complications.[35] Apart from this, glycation induces compositional and structural changes in LDL. Glycated LDL interacts with platelets leading to the development of vascular complications in diabetes by altering platelet aggregation, platelet nitric oxide production, intracellular Ca^2+^ concentration, activities of Na^+^-K^+^ and Ca^2+^ ATPases.[36]

Increased glycation of apolipoproteins may play a role in the accelerated development of atherosclerosis in diabetes and altered activity of glycated LDL receptor contributes for hyperlipidemia. In addition, glycation of liporoteins may also generate free radicals increasing oxidative damage to the lipoproteins themselves. Glycoxidation and browning of sequestered lipoproteins may further enhance their atherogenicity. The more severely modified (glycoxidized) lipoproteins in vessel walls may behave as more potent antigens than less modified particles found in the plasma stimulating the *in situ* formation of atherogenic immune complexes.[37]

The most characteristic lipid abnormality is hypertriglyceridemia with associated increase in plasma cholesterol. Elevated plasma triglyceride concentration is seen in type1 DM and type 2 DM either due to triglyceride over-production and /or underutilization. Lipoprotein lipase activity is markedly impaired, besides, a significant improvement in LDL internalization and degradation suggesting that chemical modification of LDL particle like nonenzymatic glycation of LDL itself might result in its increased incorporation in the arterial wall via a receptor independent pathway. Studies have strongly suggested an inverse relationship of HDL cholesterol with atherosclerosis to be independent of other lipid abnormalities.[38] Insulin has important effects on key steps in the metabolism of lipids and lipoproteins, and alterations in lipid metabolism are common in diabetic population.[28]

HDL cholesterol, the smallest of the lipoprotein species containing approximately 20% cholesterol ester and very little triglyceride is strongly and independently related to CHD. But, unlike LDL, the relationship is inverse, a low HDL level being an important predictor of CHD and high HDL level protecting against CHD.[39] A decrease in HDL turnover has been shown in diabetes. Some reports revealed that non-enzymatic glycosylation of HDL accelerates its catabolism in guinea pigs and the same mechanism might be responsible for the low levels of HDL in diabetic rats observed in the present study.[40]

Other studies also revealed that glycated HDL clearance is accelerated from the circulation in contrast to the effect with glycated LDL whose catabolic rate is reduced. The accelerated clearance of HDL was seen even with mild glycation and was suggested as a contributing cause of low plasma levels of HDL in diabetic patients and therefore works as another factor underlying increased risk of atherosclerotic disease in diabetic patients.[41]

In the present study, cholesterol, triglycerides and free fatty acids were brought down significantly by mulberry feeding in diabetic rats. This effect could be partly due to the control of hyperglycemia. Elevated LDL, VLDL and decreased HDL cholesterol concentrations in diabetic rats appear to be markedly altered favorably by mulberry supplementation. All the lipid abnormalities developed in STZ-diabetic rats were effectively countered by feeding mulberry leaf powder. Certain specific phytochemicals such as β sistosterol,[42] phenolics,[43] flavonoids,[44] saponins[45] and fiber[46] might be playing a role in rectifying the abnormalities. The precise mechanism underlying this effect appears to be complex. However, most of these compounds were reported to inhibit absorption of lipids from the intestines. At the same time, increased fecal bile acid excretion[47] in diabetic group treated with mulberry in the present study, reflects the conversion of cholesterol to bile acids and is a favorable feature reducing cholesterol induced risks probably by influencing either the activity or synthesis of the key enzyme, cholesterol 7α hydroxylase involved in the formation of bile acids from cholesterol. The components of mulberry leaves might also be influencing lipoprotein associated cholesterol fractions and probably the phytocomponents exert action similar to the drugs cholestyramine, mevanolin, lovastatin and simvastatin that are used for correcting the imbalance in plasma lipoproteins in diabetes. An in-depth study related to the activity of HMG COA reductase and cholesterol 7 α hydroxylase[48] in mulberry treated rats is warranted to understand the influence of the whole and individual mulberry leaf components in lowering lipid profile.

Consumption of plant material like mulberry leaves, containing antioxidants i.e. vitamin A, C, E,[49] carotenoids,[50] polyphenols[51] and phytonutrients[52] increases the antioxidant status in human blood and tissues and these compounds are capable of modulating LDL oxidation through several mechanisms. Recent studies have demonstrated that vitamin C is more potent in preventing LDL oxidation than vitamin E, and a combination of the two vitamins is even more effective than either alone.[53] Plasma levels of vitamin C and E were reported to be higher in European population with low incidence of cardiovascular diseases. Levy *et al.*[54] observed that dietary supplementation of natural β carotene normalized the elevated LDL cholesterol oxidation and thereby reduced the risk of development of atherosclerosis in diabetes. As β carotene is the chief constituent in mulberry leaves, it can be assumed that atherosclerotic preventive role was exerted by mulberry leaves by inhibiting LDL cholesterol oxidation. Doi *et al.*[55] reported the prevention of atherosclerosis by mulberry leaves. Quercetin an aglycone of isoquercetrin present in mulberry leaves inhibited the formation of conjugated dienes and TBARS by copper induced oxidative modification of rabbit and human LDLs. Similarly mulberry leaf butanol extract (MLBE) and isoqercitrin also inhibited the oxidation of LDL suggesting that mulberry leaves can inhibit the oxidative modification of LDL.[55]

From this experimental data, it is evident that mulberry leaves efficiently regulated blood glucose in diabetic rats and very efficiently ameliorated lipid abnormalities associated with diabetes in STZ-diabetic rats by virtue of various essential antioxidant, antidiabetic compounds and phytonutrients. The synergistic role played by these compounds is attributed to the protection of diabetic rats against lipid abnormalities. Further pharmacological and biochemical investigations are underway to elucidate the mechanism of the hypolipidemic effect of *Morus indica* L.cv Suguna leaves.
